# Connecting biospecimens and data: a scoping review-informed conceptual framework for digital infrastructure in biobanking and genomic research in low- and middle-income countries

**DOI:** 10.3389/fpubh.2026.1835421

**Published:** 2026-05-22

**Authors:** Anna Michalska-Falkowska, Karine Sargsyan

**Affiliations:** 1 Biobank, Medical University of Bialystok, Bialystok, Poland; 2 Cedars-Sinai Medical Center, IBD Institute and OncoBiobank, Los Angeles, CA, United States; 3 Office of Vice Rector for Research, Medical University of Graz, Austria; 4 Department of Digital Medicine and AI, Yerevan State Medical University, Yerevan, Armenia; 5 Department of Medical Genetics, Yerevan State Medical University, Yerevan, Armenia

**Keywords:** biobanking, data governance, digital health infrastructure, genomic research, health equity, low- and middle-income countries, precision medicine

## Abstract

**Background:**

Integrating biobanking and genomic research into health systems in low- and middle-income countries (LMICs) provides considerable opportunity to advance precision medicine and promote global health equity. However, persistent structural disconnect exists between the physical infrastructure for biospecimen collection and digital frameworks required to generate, manage, and share associated data. This biospecimen-data divide excludes LMIC populations from the benefits of genomic research and increases community vulnerability to extractive data policies and dependency on high-income-country (HIC) partners.

**Objective:**

This conceptual analysis aims to: (1) deconstruct the biospecimen-data divide by describing its core elements and interrelations; (2) introduce the DIGS (Digital Infrastructure, Interoperability, Governance, and Skills) Model as a framework for reconceptualizing biobanking as integrated digital enterprise; (3) examine how decisions regarding digital infrastructure may reproduce or reduce health inequities in LMIC settings.

**Methods:**

A critical scoping review was conducted using the Arksey and O’Malley framework and reported in line with PRISMA-ScR. Five databases—PubMed, Scopus, Web of Science, Embase, and Global Index Medicus—were systematically searched, yielding 2,390 records. After deduplication (*n* = 880), title and abstract screening (*n* = 1510), and full-text review (*n* = 296), 154 studies were included in the final synthesis. These included peer-reviewed articles, policy documents, and grey literature on biobanking, digital health infrastructure, and data governance in LMIC contexts.

**Results:**

Four interdependent structural gaps underpin the biospecimen-data divide: (1) infrastructure gap, reflecting constraints on data generation and flow; (2) interoperability gap, arising from incompatible systems that create data silos; (3) human-capital gap, marked by limited availability of personnel with combined laboratory and data stewardship expertise; (4) governance gap, defined by weak ethical and regulatory structures. Together, these gaps reveal deeper asymmetries between HICs and LMICs. The DIGS Model reframes biobanking as a cyclical, equitable process requiring coordinated investment across all four dimensions.

**Discussion:**

The DIGS Model challenges the prevailing infrastructure-first paradigm, which prioritizes physical assets over digital capabilities. It advances partnership models that center LMIC leadership in data governance, redefining sustainability as the cultivation of local capacity to generate, interpret, and control data. The framework offers researchers, funders, and policymakers a shared diagnostic tool for bridging the biospecimen-data divide without reinforcing existing dependencies.

## 1 Introduction

### 1.1 The genomic revolution and its potential for LMICs

Recent advances in genomic science and technology have helped us understand the causes of many diseases, inspired new approaches in genomic medicine, and given us tools for precision public health at the population level. Infectious, non-communicable, and neglected tropical diseases are especially common in Low- and Middle-Income Countries (LMICs), where genomics could help reduce health inequalities and suffering. Although the cost of sequencing a genome has dropped from $95 million in 2001 to less than $1,000 in 2022, resource-poor settings still face major challenges with infrastructure and capacity. Those challenges are the main focus of this study (1–3).

Initiatives like the Human Heredity and Health in Africa (H3Africa) Consortium have helped promote genomic research for African populations. Current efforts concentrate on building capacity and guaranteeing fair data sharing (4, 5).

Pathogen genomics has shown strong potential for field surveillance and outbreak management, though rapid technical responses have not consistently produced lasting digital infrastructure. Gaps in data generation and governance show that technical progress alone, without investment in digital systems, governance, and skills, leads to fragile and unequal results (6). To completely realize the benefits of genomics for inclusive health and meet the United Nations Sustainable Development Goal 3, *Good Health and Well-being*, we need not only laboratory capacity but also digital systems that link biospecimens to data (7, 8).

### 1.2 Biobanks as critical infrastructure for digital health in LMICs

Biobanks are essential to the genomic and digital health sector. They systematically collect, process, store, and share biospecimens along with related clinical and molecular data. Rather than being static archives, biobanks act as active hubs that connect local communities, healthcare providers, and researchers (9). In LMICs, where health systems encounter overlapping challenges from infectious diseases, chronic conditions, and new threats, biobanks are key to using digital technologies for fairer health outcomes, as long as the technological side of this infrastructure is well developed (10).

Digital tools have become more common in biobanking, with systems such as biobank information management systems (BIMS) and laboratory information management systems (LIMS) helping automate biospecimen tracking, reduce errors, and improve data quality (11, 12). Cloud systems and virtual biobanks could enable institutions to search distributed repositories without moving physical samples, but in LMICs, these solutions are still new and rely on stable internet connections (13, 14). Importantly, digital progress is more than just using new devices. It means creating workflows so that each biospecimen is treated as a data-rich object from collection to analysis and reuse, following the biospecimen-as-data-carrier idea (15). Building local control over these digital systems is important to make sure the benefits from research reach the communities where the samples come from (10). Even with new technology, biobanks in LMICs still face big challenges that highlight both their importance and their vulnerability.

Infrastructure problems, like unreliable electricity and internet, slow down digitization in many LMICs. A survey by Mendy and colleagues found that about 55% of biobanking facilities in 23 LMICs had uninterrupted power (16). But this average hides big differences: facilities in upper-middle-income countries and large cities often have better reliability, while those in rural or low-income areas sometimes have less than 30% reliable power (15, 16). Various regions have seen small improvements, especially where solar backup systems are used, but there is still not much updated data across all LMICs (17). Limited budgets and not enough skilled staff make things harder, often forcing reliance on outside funding and cross-border partnerships. Gaps in ethics and regulations, especially concerning data privacy, can threaten participant trust and data security, especially within diverse cultural settings in which informed consent may be difficult (18).

Recent projects show that biobanks are playing a bigger part in promoting health equity. In Latin America, expanding biobanking has focused on involving communities and meeting local health needs, while also tackling funding and regulatory challenges to improve research on regional diseases (19). The Pan-African Biobanking Network’s 2024–2025 survey found progress in building repositories across Africa, even though there are still issues with standardization and visibility. These are being addressed through fair partnership models (20). The FIND Integrated Biobank Network shows how networked governance can boost efficiency and help LMICs prepare for pandemics (21). Together, these efforts show that the main barriers are not simply technical, but are deeper structural issues: the gap between biospecimens and data, which need coordinated investment in infrastructure, governance, and skills.

### 1.3 The central problem: the biospecimen-data divide

There is a clear imbalance in biobanking investments in LMICs. Most resources and genomic data examination capabilities are found in wealthier countries and institutions (22). This asymmetry has been characterized in the literature as a pattern of extractive genomic research: Researchers describe this as extractive genomic research, where biospecimens and data are taken from LMIC populations and sent to high-income countries (HICs), but local communities do not receive equal benefits, analytical skills, or control (23–25). While a lot is spent on physical infrastructure like freezers, lab equipment, and storage, digital systems regularly get much less attention (26). This focus misses important needs, such as systems for managing clinical data, platforms that connect biospecimens to health outcomes, ethical data-sharing structures, and trained staff to run these digital tools (27). As a result, biobanks in LMICs often focus on collecting and storing samples but have trouble using digital tools effectively. This leads to less useful research and ongoing health inequities (15, 28). This situation is known as the “biospecimen-data divide,” which describes the gap between collecting and storing samples and being able to use the related data for scientific progress (29).

In many LMICs, the biospecimen-data divide worsens due to weak infrastructure, which makes it hard to use key technologies such as BIMS and LIMS. Old IT systems from different projects make it difficult to connect data across repositories and link with health records or omics datasets (28). If biospecimens are not linked to clinical, demographic, or genomic data, they have little value for developing treatments or public health programs, turning them into unused collections (30). Sending biospecimens from LMICs to high-income countries for analysis, without fair sharing of data or benefits, increases global inequalities. For example, in Armenia, limited bioinformatics skills and regulatory issues prevent local data use and participation in international projects (31). Some new projects, like the DxConnect Virtual Biobank and drone-based sample transport in Rwanda (32, 33) show ways to close this gap with digital tools and better logistics. However, ongoing problems with funding (such as Armenia’s R&D spending at only 0.32% of GDP) (31) and lack of regulatory coordination still slow progress. In One Health approaches, this imbalance makes LMICs more vulnerable during disease outbreaks, as poor data sharing and inconsistent information weaken surveillance and response, even though these countries often face higher disease burdens (28). To close the biospecimen-data divide, LMICs need targeted investments in digital infrastructure, training for local experts, and ethical guidelines that match international standards like ISO 20387 and GDPR (34).

### 1.4 Heterogeneity within the LMIC category: LIC, LMIC, and UMIC distinctions

The category “low- and middle-income countries” encompasses 129 economies spanning an extraordinary range of income levels, state capacities, health system maturities, and digital infrastructure landscapes. The World Bank’s current income classifications distinguish between low-income countries (LICs) with Gross National Income (GNI) per capita ≤ USD 1,135, LMICs: GNI per capita USD 1,136–4,495, upper-middle-income countries (UMICs): GNI per capita USD 4,496–13,935, and HICs: GNI per capita >USD 13,935 (35). While this manuscript uses “LMICs” as a shorthand for the full range of non-HICs settings, the DIGS Model’s relevance and priority dimensions differ meaningfully across this spectrum.

### 1.5 Aims

This conceptual analysis argues that to bridge the gap between biospecimens and data in biobanking within LMICs, we need to shift our view of biobanks. Instead of seeing them mainly as storage facilities, we should see them as digital enterprises that connect biospecimens with advanced data systems (12, 22). We suggest that local researchers, policymakers, and funders should work together to reorganize biobanking strategies and support fair knowledge creation (23). To support this idea, the analysis has three parts. First, it breaks down the biospecimen-data gap by looking at key issues like data differences, errors before analysis, and problems with systems working together. Second, it introduces the DIGS Model (Digital Infrastructure, Interoperability, Governance, and Skills), which brings these parts together inside a framework that can work in settings with fewer resources. This model is based on data governance in digital health and points out the need for guidelines that protect privacy and security. Third, the analysis looks at how this new approach could affect research methods by fostering the use of AI to make better use of biospecimens. Finally, it discusses how virtual biobanks can help make global health research more inclusive and reduce gaps in biomedical progress (36, 37).

## 2 Methods

### 2.1 Methodological framework

We conducted a critical scoping review using the five-stage framework by Arksey and O’Malley (38) updated by Levac et al. (39), and followed the PRISMA-ScR checklist (40). We chose a scoping review because our aim was to map concepts and build a framework. The review focused on three main research questions: (1) What are the main barriers to adding digital infrastructure to biobanking in LMICs, and how are these barriers connected? (2) What frameworks, governance models, and technical standards have been suggested or used to address these challenges? (3) How can a single framework bring together infrastructure, interoperability, governance, and skills to support fair biobanking in settings with limited resources?

### 2.2 Search strategy, database selection, screening, and eligibility criteria

We systematically searched five electronic databases: PubMed/MEDLINE, Scopus, Web of Science, Embase, and Global Index Medicus, covering the period from January 2010 to January 2026. We also searched targeted grey literature, including policy documents, technical standards, and reports from organizations such as World Health Organization (WHO), the World Bank, the H3Africa Consortium, Global Alliance for Genomics and Health (GA4GH), ISBER, Biobanking and BioMolecular Resources Research Infrastructure – European Research Infrastructure Consortium (BBMRI-ERIC), the African Union Commission, the Convention on Biological Diversity (Nagoya Protocol), and the FIND Biobank Network.

We built our search strings using Medical Subject Headings (MeSH) for PubMed, Emtree terms for Embase, and similar controlled vocabulary for Scopus and Web of Science. We also included free-text terms and combined them with Boolean operators. The full search strings for each database are provided in Supplementary file S1.

Two reviewers (AMF and KS) independently screened all retrieved records using Rayyan systematic review software (41). Title and abstract screening was conducted in a blind mode, in which Rayyan concealed each reviewer’s decisions until both had completed their screening of a given record. At the title and abstract stage, records were classified as include, exclude, or maybe. All records classified as maybe by either reviewer proceeded to full-text review. Disagreements between reviewers at both stages, defined as one reviewer selecting include, and the other selecting exclude, were resolved through synchronous discussion until consensus was reached. To be included, studies had to cover at least one of these topics: biobanking, genomic data management, digital health infrastructure, data governance, health informatics, precision medicine, or capacity-building in LMIC settings. Some studies from HICs were included if they provided important technical standards, theoretical frameworks, or other directly relevant ideas. Eligible publications included peer-reviewed research, systematic and scoping reviews, conceptual articles, policy analyses, consensus statements, official guidelines, and grey literature from recognized international organizations. Only English-language reports were included. We excluded reports that focused solely on biobanks in HICs without applicable lessons for LMICs, addressed only non-human biospecimens, were conference abstracts without an accompanying full report, or were opinion pieces without supporting evidence.

### 2.3 Data extraction, charting, and synthesis

We extracted data using a standard charting form (Supplementary file S2) that included: (1) author(s) and year, (2) country or region of study, (3) study design or document type, (4) main topic area, (5) key findings or arguments about the biospecimen-data divide, and (6) specific relevance to DIGS Model dimensions. Data were extracted into a standardized Microsoft Excel-based charting form (Supplementary file S2) by the lead reviewer (AMF). A random 25% sample of extracted records (*n* = 38) was independently verified by the second reviewer (KS) to assess extraction consistency. Any discrepancies identified in this verification sample were discussed and resolved by consensus before the verified extraction was accepted as final. We used Rayyan (41) to remove duplicate records across databases.

We analyzed the extracted data using thematic analysis following Braun and Clarke (42), with adjustments for literature-based evidence. We identified themes directly from the records, then mapped them to the four proposed DIGS Model dimensions. Our team went through several rounds of coding, developing themes, and refining concepts. The DIGS Model was developed from this process, not applied afterward. Its four dimensions were based on patterns of gaps and solutions found in the literature, and we formalized and named them during our research.

We used scoping review methods and did not formally assess the quality of individual studies, since our goal was to map concepts rather than combine effect estimates (38, 39). To check source credibility, we used a three-tier classification for all 154 studies. Tier 1 includes primary empirical evidence from peer-reviewed research conducted in, or directly relevant to, LMIC settings. Tier 2 covers policy and normative documents from major international organizations such as WHO, ISBER, GA4GH, ISO, and H3Africa. Tier 3 consists of conceptual, theoretical, and interpretive literature that provides analytical context. In the manuscript, we link claims to their evidence tier and clearly state when empirical claims are based only on Tier 3 records.

## 3 Results

### 3.1 Search results and source selection

**Figure 1:**
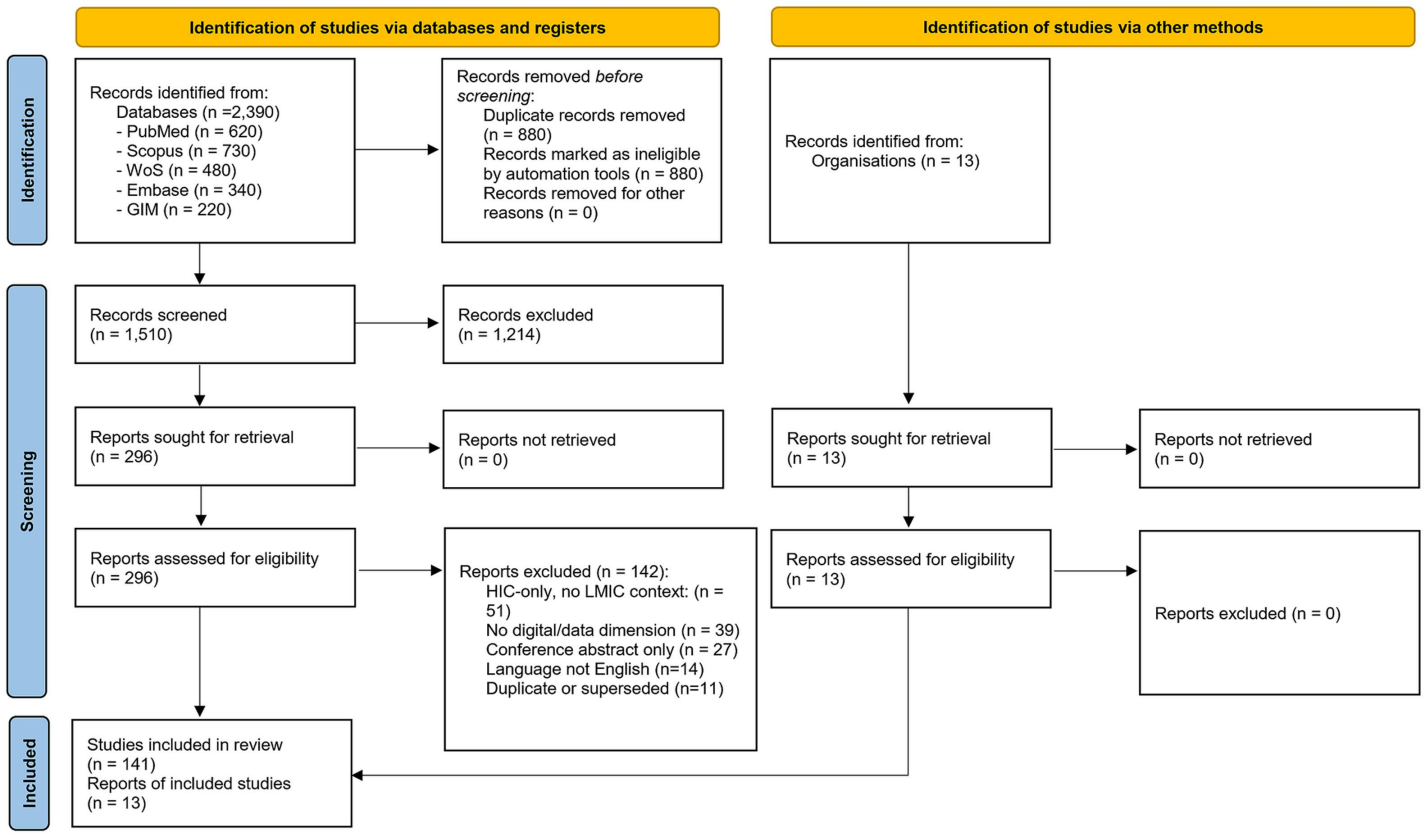
PRISMA-ScR flow diagram for the DIGS Model scoping review. We identified records by searching five electronic databases from January 2010 to January 2026: PubMed/MEDLINE (620), Scopus (730), Web of Science Core Collection (480), Embase (340), and Global Index Medicus (220), for a total of 2,390 records. After removing 880 duplicates, 1,510 unique records were screened by title and abstract. We excluded 1,214 records because they were outside the topic, not focused on LMICs, or did not include a digital or data aspect. The final 154 studies included 59 original research articles, 36 narrative reviews, 22 perspectives and commentaries, 15 policy analyses and technical standards, 10 systematic and scoping reviews, and 6 primary grey literature documents. By DIGS dimension, there were 51 for Digital Infrastructure, 33 for Interoperability, 40 for Governance, 11 for Skills, 18 for background context, and 1 cross-cutting. Most of the evidence comes from sub-Saharan Africa, which is noted as a limitation in Section 6.2. GIM stands for Global Index Medicus; HIC for high-income country; LMIC for low- and middle-income country; and PRISMA-ScR for preferred reporting items for systematic reviews and meta-analyses extension for scoping reviews.

The systematic search found 2,390 records from five databases: PubMed/MEDLINE (620), Scopus (730), Web of Science (480), Embase (340), and Global Index Medicus (220). After removing 880 duplicates (36.82%), we screened 1,510 unique records by title and abstract. During title and abstract screening, we excluded 1,214 records because they did not focus on biobanking in LMICs, lacked relevant topics like digital infrastructure, interoperability, data governance, or skills, or did not meet the inclusion criteria. We reviewed 296 full reports for eligibility assessment. After full-report assessment, 154 studies from the database searches met all inclusion criteria. Figure 1 shows the PRISMA flow diagram (43).

The 154 included studies comprised original research articles (*n* = 59), narrative reviews (*n* = 36), perspectives and commentaries (*n* = 22), policy analyses and technical standards (*n* = 15), systematic and scoping reviews (*n* = 10), and primary grey literature documents (*n* = 6). By topic, sources covered digital infrastructure (*n* = 51), governance (*n* = 40), interoperability (*n* = 33), skills (*n* = 11), background context (14), and one cross-cutting study, as summarized in Table 1.

**Table 1:** Summary of included studies by document type, DIGS dimension, geographic focus, and evidence tier.

Characteristic	Category	*n* (%)
Document type	Original research	59 (38%)
	Narrative review	36 (23%)
	Perspective/Commentary	22 (14%)
	Policy analysis/Technical standard	15 (10%)
	Systematic/Scoping review	10 (7%)
	Grey literature	6 (4%)
Primary DIGS dimension	D1 Digital Infrastructure	51 (33%)
	D2 Interoperability	33 (21%)
	D3 Governance	40 (26%)
	D4 Skills	11 (7%)
	Background context	18 (12%)
	Cross-cutting	1 (1%)
Geographic focus	Sub-Saharan Africa	35 (23%)
	Global/Multi-region	66 (43%)
	Latin America	4 (3%)
	South/Southeast Asia	7 (5%)
	Middle East/Eastern Europe	5 (3%)
	HIC (comparator only)	37 (24%)
Evidence tier	Tier 1 (LMIC empirical)	91 (59%)
	Tier 2 (Policy/normative)	24 (16%)
	Tier 3 (Conceptual/theoretical)	39 (25%)

HIC-focused sources were included selectively as technical or conceptual comparators; they are not counted as primary LMIC evidence. Full extracted data for all 154 studies are provided in Supplementary file S2.

### 3.2 Deconstructing the biospecimen-data divide: four interconnected gaps

**Figure 2:**
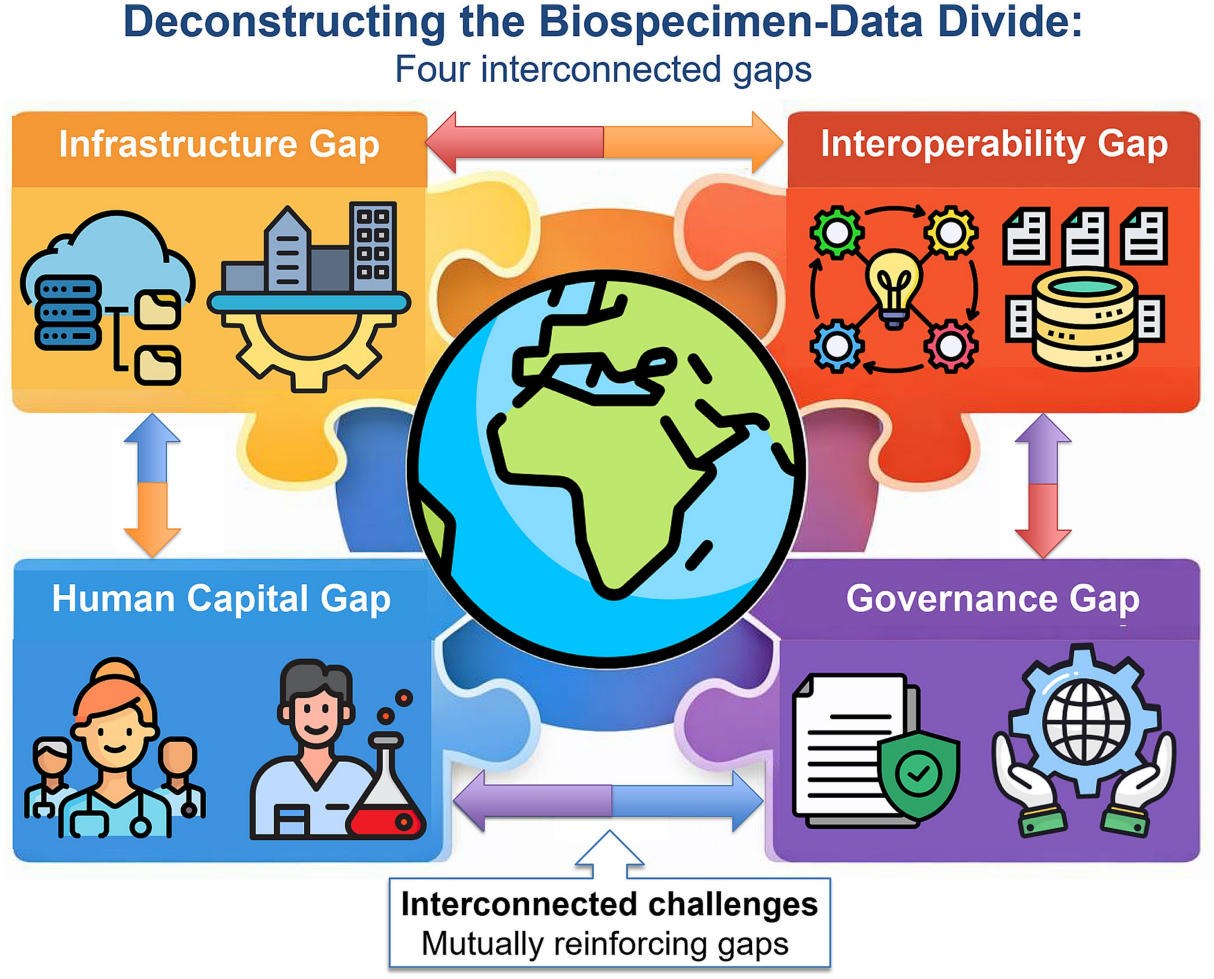
The biospecimen-data divide: four interconnected structural gaps in LMIC biobanking. The diagram shows four main gaps that together form the biospecimen-data divide in biobanking in low- and middle-income countries (LMICs). The infrastructure gap (top left) encompasses material problems such as unreliable electricity, poor connectivity, and insufficient computing hardware, all of which make it hard to generate data when collecting biospecimens. The interoperability gap (top right) refers to the fragmentation of health information systems that prevents them from sharing or using data, so biospecimens cannot be linked to ongoing clinical, genomic, and phenotypic records. The human capital gap (bottom left) refers to the shortage of people with the right mix of lab science, data management, bioinformatics, and research ethics skills needed to run digital biobanking systems. This is made worse by patterns that keep trained experts in high-income institutions. The governance gap (bottom right) points to the lack of strong national data protection laws, effective ethical review systems for genomic data sharing, and the use of international frameworks such as the Nagoya Protocol and the CARE principles for indigenous data governance. The arrows in the diagram show that all four gaps are connected: if one area is weak, it holds back the others. This is why single solutions do not fix the biospecimen-data divide. CARE stands for collective benefit, authority to control, responsibility, and ethics. LMICs means low- and middle-income countries.

The biospecimen-data divide comprises several distinct yet interconnected gaps. Seeing these gaps as related, rather than as separate issues, is important for finding effective solutions. Figure 2 shows four main gaps: infrastructure, interoperability, human capital, and governance. Together, these form the foundation of the divide.

### 3.3 The infrastructure gap: material conditions for data generation

The infrastructure gap refers to the physical conditions that help or hinder the collection of data from biospecimens. In many low- and middle-income countries, these conditions are often unreliable and vary by region (44). In many rural medical centers in sub-Saharan Africa and Southeast Asia, electricity can be unreliable or missing, making on-site data entry difficult (45, 46). Internet access, when available, is often too slow, costly, or unstable to transfer large genomic files, especially in areas that rely on mobile networks instead of fiber optics (47). Devices like tablets, laptops, and servers may also be in short supply, outdated, or not well-maintained (11, 15).

These physical challenges have many effects. When data entry at the collection site is not possible, staff must use paper forms, which can lead to mistakes and delays. Electronic health records, if available, often work as separate systems instead of being connected. This creates a divided data environment where biospecimens and their clinical data are kept in different, unconnected places (48). The infrastructure gap is more than simply a technical problem; it reflects more profound structural inequalities. The same global economic differences that place most biotechnology industries in HICs also affect how digital infrastructure is spread around the world (49). LMICs are expected to join a data-driven scientific field without the basic infrastructure that HICs already have (48, 50).

### 3.4 The interoperability gap: the problem of fragmented systems

Even when infrastructure is in place, another problem appears: different data systems often cannot communicate with each other. Interoperability, which means information systems can exchange and use data, is essential for biobanking to reach its full potential (51). Biospecimens are most valuable when they are connected to long-term clinical data, treatment results, and other health information (12, 52).

In LMICs, health information systems are frequently fragmented (53). One patient might have records in several separate systems, such as a tuberculosis registry, an HIV clinic database, a maternal health program, and a research biobank (54, 55). Even when unique patient identifiers exist, they may not be used consistently (56). Data standards also differ between systems, which makes integration difficult and time-consuming (57). Without shared standards like HL7 FHIR, LOINC, or SNOMED, exchanging data is still a manual and error-prone process (58–60). The BBMRI-ERIC provides a model for large-volume data harmonization. Its MIABIS (Minimum Information about Biobank Data Sharing) standard defines the core dataset needed to make biobank samples easy to find and use across institutions, and serves as a benchmark for measuring interoperability gaps in LMICs (61, 62).

This disintegration has both technical and epistemic dimensions. Technically, it requires investments in data standards, application programming interfaces (APIs), and system architecture (63). On the knowledge side, it provokes questions about which data are important enough to collect and connect. Deciding which data elements to standardize and whose standards to use is not a neutral process; it reflects specific scientific and political priorities (64). As a result, these systems shape what information is noticed and acted on in research communities (65). Also, when terminology and coding differ between platforms, analysts must spend extra time and resources matching data formats before they can combine information in a meaningful way (66).

### 3.5 The human capital gap: the missing cadre of data professionals

A third important gap is the need for skilled people inside digital infrastructure. Biobanking today needs staff who can combine lab skills for handling biospecimens, data science skills for managing multifaceted datasets, and ethical skills for issues like consent and governance (67). These “bio-data stewards” play a key role in turning biospecimens into useful knowledge (68). To develop this talent, organizations should create training programs that connect traditional lab work and data management (36, 69).

In LMICs, there are not enough of these professionals (70). Training has usually focused more on lab techniques than on data management, and career paths for data experts in health and research are often not well defined (71, 72). As a result, local teams commonly rely on outside partners for data analysis, which can create ongoing dependencies and reduce local control over research (34, 73). Missing clear career options, many trained staff leave for better opportunities in international health programs instead of staying to support local research (74).

This gap in skilled people matters because it affects who gets to ask questions about the data. When most data analysis happens in HICs, research commonly follows outside priorities instead of local health needs (75). Closing this gap is important for both technical progress and fairness in knowledge (76). If regional institutions build their own expertise, they can guide research and make sure health solutions fit their communities (77). To do this, local researchers need to be involved at every stage of research (78). Also, LMIC leaders should set research priorities so that data analysis assists local policy and health system improvements (79).

### 3.6 The governance gap: the ethical and regulatory vacuum

The fourth gap is about the governance frameworks needed to guide how data is collected, stored, shared, and used. In many LMICs, national data protection laws are either missing, outdated, or not properly enforced (80, 81). Across Africa, the African Union Data Policy Framework sets out principles for data sovereignty, cross-border data flows, and the management of health and genomic data among AU member states. This framework delivers a structure for national biobanking governance (82). However, most African LMICs have not fully put these continental policies into practice at the national or institutional level. For instance, there may be no specific laws on genomic data, and institutional review boards commonly lack genomics expertise. Ethical review committees may also be unfamiliar with the complex issues involved in genomic data sharing, dynamic consent, and international data transfer (83).

These issues are not simply theoretical. For example, the Havasupai Tribe case in the United States showed what can go wrong when blood samples collected for diabetes research were later used without permission for studies on schizophrenia and population migration (84). During the 2014–2016 West African Ebola outbreak, there were also disputes about exporting blood samples, demonstrating the ongoing conflict between quick pandemic response and fair data governance (85). When biospecimens were sent to labs in wealthier countries for analysis, people questioned whether the data and benefits would return to the communities and researchers who provided the samples (86).

The governance gap affects several levels. On the individual level, it can make consent meaningless, turning informed permission into just a formality (87). On the community level, it damages trust between communities and research institutions, which can discourage people from taking part in future biomedical research (88). On a larger scale, it keeps LMICs in the role of supplying raw materials, like biospecimens, instead of producing valuable results such as scientific discoveries, intellectual property, or commercial products (89–91). This disproportion is made worse by unclear rules about benefit sharing, which often do not protect researchers and participants in resource-limited settings from being exploited (92).

To close this gap, governance needs to go beyond just following rules and ticking checkboxes. Instead, it should focus on fairness, reciprocity, and shared control. Internationally, the Nagoya Protocol on Access and Benefit-Sharing sets legal requirements for countries and researchers who use genetic resources. It calls for prior informed consent, agreed terms, and fair sharing of benefits with the country where the resources come from. Even though the Nagoya Protocol is important for collecting and transferring biospecimens in LMICs, it is not well included in most biobanking governance frameworks there. Many international genomic research partnerships also do not clearly refer to its rules, which is a gap that the DIGS Model’s Governance dimension needs to address (93).

For governance in LMIC biobanking to be effective, it should be developed together with local partners, address community concerns, and follow principles like GA4GH and the CARE Principles (Collective Benefit, Authority to Control, Responsibility, Ethics) for Indigenous Data Governance. These steps help guarantee fairness and local control (94–96). Without these changes, the governance gap will keep harming both the ethics and the quality of genomic research within resource-limited settings.

### 3.7 The interconnections between gaps

These four gaps are connected and strengthen each other (97). Poor infrastructure makes it hard to set up interoperable systems, which leads to more segregated data silos and blocks smooth information sharing. This trend is clear when comparing different regions. Countries that invest more in digital systems, like Rwanda with its integrated health information system, show better interoperability than those with less developed infrastructure (98). Still, the situation is complicated. Good infrastructure by itself is not enough for interoperability without also investing in standards and workforce training (99).

When systems are not interoperable, it is harder to build local data expertise. Without standard technical frameworks, it is also difficult to provide practical training for a workforce skilled in advanced data management and analytics (34). A lack of local expertise makes it tough to set up and maintain strong governance, and weak governance in turn discourages investment in infrastructure (99). This creates a cycle where limited efforts to build capacity slow down national development plans and delay the growth of strong data ecosystems (100). The gap between biospecimen data and other data is made up of several related problems. To solve this, it is important to use universal, stable identifiers that establish lasting links between biospecimen catalogues and genomic databases (101). These technical procedures should be supported by clear institutional rules that make data curation a main priority, guaranteeing that trained and supported staff consistently follow standard workflows (102, 103).

### 3.8 Toward a new conceptualization: biobanking as an integrated digital Enterprise

The analysis above shows that current ideas about biobanking fall short. The main approach, often called the “physical repository model,” sees biobanks mostly as places to store biospecimens (36). In this view, success depends on how many samples are stored, how well they are preserved, and how efficiently they are distributed (104, 105). Digital systems, if considered at all, are seen as secondary rather than central to the biobank’s purpose.

In contrast, an integrated digital enterprise model focuses on the smooth movement of biospecimens and their related long-term data, turning static collections into active parts of a global research network (106). In this model, data processes are included from the beginning, and there is a focus on responding to new technologies. By using tools like artificial intelligence (AI) and blockchain with strong oversight, this approach tackles ongoing problems like tracking samples and keeping collections useful for science (26). In the age of precision medicine, this integration lets biobanks support high-speed sequencing, machine learning, and links to health records, which leads to insights at the population level (107). Each biospecimen is seen as a source of information, from DNA to physical traits. Making biobanking “FAIR”: making biospecimens and their data “findable, accessible, interoperable, and reusable”is part of this goal (108). This change means moving away from separate data management to systems that work together, making it easier to track samples and share data across institutions (26, 109). This adjustment also means using a “fit-for-purpose” quality approach, where careful documentation of each biospecimen’s history helps prepare for future technology (104).

Today, with genomics and precision medicine, a biobank that lacks digital systems cannot take part in, and may be left out of, the data-heavy research networks changing global health science (109). More importantly, the physical repository model hides the fact that biobanks are, and have always been, data organizations (110). By moving from a focus on samples to an emphasis on data, institutions can bring together different types of information, such as genomic, clinical, and pathology records (12). This change can help connect the gap between traditional biospecimen collection and the needs of precision health, where biospecimens are used for advanced analytics and insights at the population level (107).

### 3.9 Biospecimens as data carrier

Every biospecimen serves as a repository of critical data, encompassing the donor’s identity, the precise time and location of collection, relevant clinical context, and the biospecimen’s processing history. These data elements are not ancillary to the biospecimen; rather, they are key to its scientific significance. A biospecimen lacking such associated data cannot be distinguished from any generic biological tissue or blood sample (111). It is the presence of complete data that transforms a biospecimen into scientifically valuable evidence (112).

Recognizing biospecimens as fundamental data carriers has substantial impact on biobanking and biomedical research. The quality of a biobank cannot be determined solely by the physical state of its stored biospecimens; it is equally dependent on the quality and completeness of the associated data (113). Thus, investments in biospecimen collection must be paralleled by equivalent investments in systematic data acquisition and management (114). Furthermore, the ethical principles regulating data—such as privacy, confidentiality, and participant autonomy—must be rigorously applied to the biospecimens themselves, considering their dual role as physical and informational entities (115). This fundamental change toward integrated provenance requires transitioning from siloed, local data practices to a standardized, cross-organizational model that provides the non-repudiation and long-term interoperability of biological resources (116). Such harmonization efforts necessitate the generation of robust provenance models that link the sample’s lifecycle directly to the clinical and omics data generated during downstream research (117).

### 3.10 The data lifecycle in biobanking

To view biobanking as a digital enterprise, it is important to consider the whole data lifecycle, including data generation, processing, storage, analysis, sharing, and reuse (118). Each stage brings its own challenges and opportunities, especially in LMIC settings, where limited resources and local factors make managing and using biobank data more difficult (30, 94).

Data generation happens at several points, starting with the consent process and continuing through clinical data entry and lab work. These steps can introduce errors, but they also offer a chance to support equity by making sure data acquisition methods fit local needs and research goals (119, 120). After data is collected, it must be carefully processed—cleaned, coded, and organized—to make sure it is accurate, consistent, and useful (121). Good data processing relies on strong technical systems and skilled staff (121, 122).

Storing data requires strong security, including privacy protection and long-term preservation (123). This step is key to keeping data reliable and available, especially where technology resources are limited. Data analysis is a turning point in the lifecycle because careful review and interpretation create new scientific knowledge. The people or teams doing the analysis, the tools they use, and the questions they ask all shape the kind of knowledge that results (124, 125).

Sharing and reusing data are key to making the most of their research value (126). The rules and systems that control data sharing decide who can access and benefit from this knowledge, bringing up important questions concerning fairness, access, and benefit-sharing in science (83, 127).

Looking at the whole data lifecycle shows that the gap between biospecimens and their data is not simply a single issue. Instead, there are many points where problems can happen, from data generation to reuse (128). These issues can affect the quality, access, and usefulness of biobank resources, especially in LMICs where difficulties are greater (22). Connecting this gap takes strong, context-aware strategies that keep data moving fluidly, securely, and fairly at every stage (34). This is essential for making biobanking research impactful, supporting reproducible studies, and making sure that the benefits of biobank knowledge are shared widely and fairly in the global research community (129).

## 4 The DIGS Model: a comprehensive conceptual framework

Building on the preceding analysis, this study introduces the DIGS Model as a conceptual framework tailored to the structural realities of biobanking in LMICs. It is important to note that these four dimensions are not exclusive to LMICs. HICs biobanking networks, such as BBMRI-ERIC member institutions, also focus on infrastructure, interoperability, governance, and skills (130). What sets the DIGS Model apart for LMICs are three features that define their unique challenges. First, the type and seriousness of each gap are very different. HICs biobanks work on improving existing systems, like speeding up data pipelines, updating LIMS, or adding cloud storage. In contrast, LMIC biobanks, especially in LICs, often lack basic infrastructure, such as reliable electricity, internet access, or functioning LIMS. Governance issues also differ: HICs focus on GDPR compliance and data-use agreements, while LMICs must address extractive research practices, put the Nagoya Protocol into practice, and create national regulations that are not yet in place. So, terms like “infrastructure” or “governance” refer to very different challenges in each context. Second, the cyclical interdependence of the four dimensions is particularly explanatory in LMIC settings because isolated single-dimension interventions have systematically failed there. Donor-funded projects have repeatedly built freezer capacity without digital systems, trained bioinformaticians who then emigrated, or established data-sharing platforms that remained unused because local staff lacked the skills to operate them (131). Third, the equity implications are inherent to the LMIC context: LMIC communities provide biospecimens and carry the risks of genomic research participation; the analytical outputs, intellectual property, and commercial benefits have disproportionately accrued to HIC institutions. The DIGS Model explicitly addresses it through the Governance dimension’s emphasis on benefit-sharing, data sovereignty, and CARE Principles implementation (96). Section 4.6 illustrates concretely how DIGS Model application translates into measurable improvements in LMIC biobanking practice.

**Figure 3:**
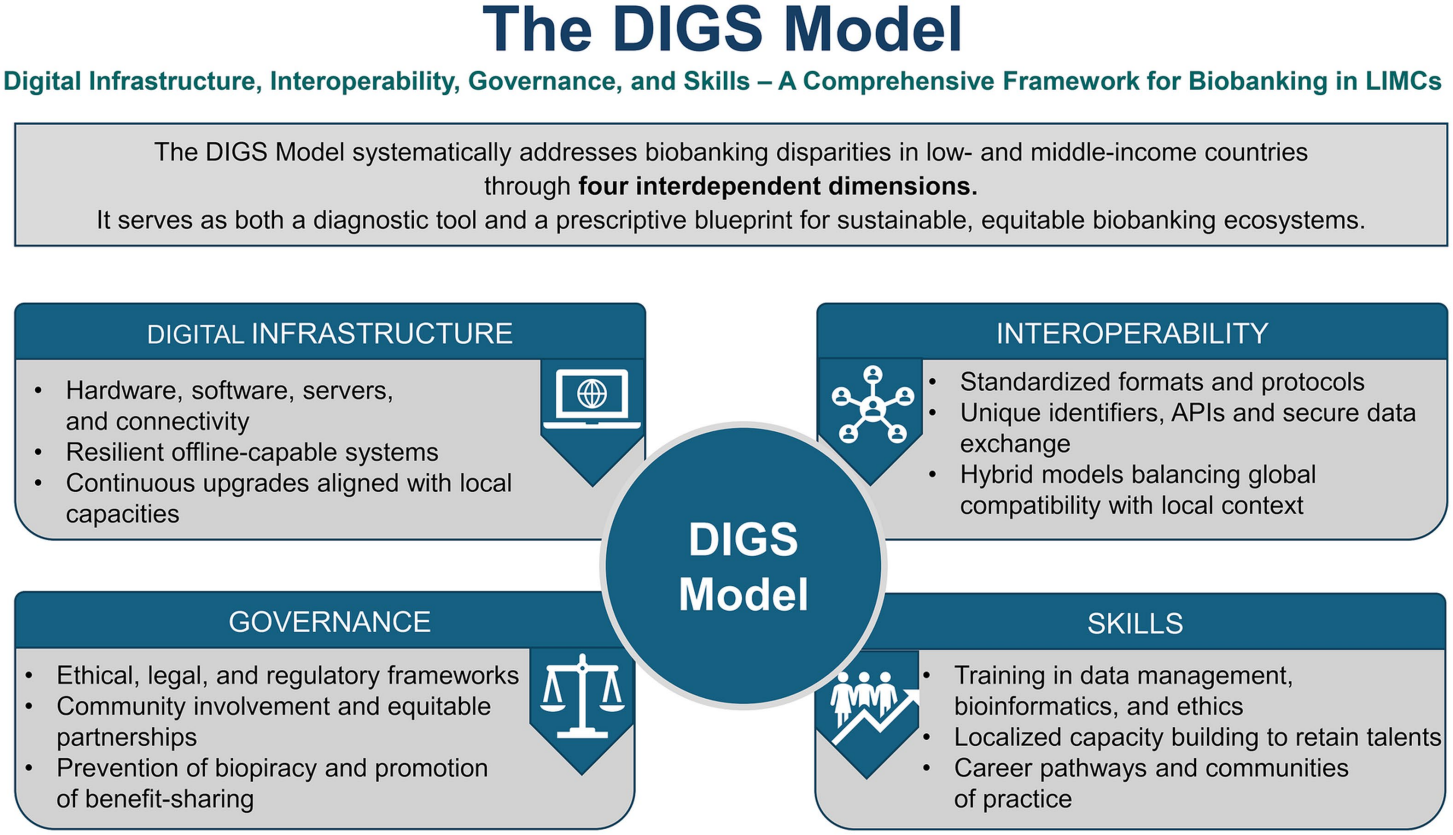
The DIGS Model: an integrated conceptual framework for digital biobanking in low- and middle-income countries. The DIGS Model suggests that for biobanking to be sustainable and fair in settings with limited resources, four connected areas must be developed together. These areas form a cycle. The first area, digital infrastructure (D1), includes the hardware, software, connectivity, and data systems needed to handle biospecimen data. It focuses on solutions that work offline, need little maintenance, and fit the needs of low- and middle-income countries (LMICs). The second area, interoperability (D2), is about using standard data formats, terms, unique identifiers, and application programming interfaces (APIs) so that biospecimen catalogues can connect across institutions, link with health records and omics data, and be found using the FAIR principles (Findable, Accessible, Interoperable, Reusable). The third area, governance (D3), covers the ethical, legal, and regulatory systems. This includes national data protection laws, ethical review processes, community involvement, following the Nagoya Protocol on access and benefit-sharing, and putting the CARE principles into practice. These steps help ensure trust, accountability, and fair sharing of benefits. The fourth area, skills (D4), is about the people needed to design, run, and manage digital biobanking systems. This includes building local career paths, creating LMIC-led training groups, and avoiding practices that take advantage of local capacity-building. The arrows in the model show that each area supports and relies on the others. To close the gap between biospecimens and data, all four areas need to be developed at the same time, not one after another or in isolation. The DIGS Model can be used to spot weaknesses in each area and to guide the design of complete solutions. API stands for application programming interface; CARE stands for collective benefit, authority to control, responsibility, ethics; DIGS stands for digital infrastructure, interoperability, governance, and skills; FAIR stands for findable, accessible, interoperable, reusable; LMIC means low- and middle-income country.

By stressing adaptability and local relevance, the DIGS Model functions as both a diagnostic tool for identifying contextual weaknesses and a prescriptive guide for multifaceted interventions, thereby supporting the development of sustainable biobanking ecosystems that promote global health equity and advance Sustainable Development Goal 3. The four-dimensional framework of the DIGS Model is presented in Figure 3.

### 4.1 Dimension one: digital infrastructure

Digital infrastructure encompasses the core materials and technological systems that support the generation, transmission, and utilization of data in biobanking operations (15, 132). This dimension includes hardware such as computing devices, servers, and networking equipment; software solutions including data management platforms and analytical tools; and connectivity elements such as internet bandwidth and mobile networks. Within the DIGS Model, digital infrastructure is conceptualized as a dynamic, iterative process demanding continuous maintenance, upgrades, and modification to progressing technological and operational demands. In LMICs, obstacles such as unreliable power supplies and intermittent connectivity are prevalent, affecting approximately 55% of biobanks according to recent surveys (15). Subsequent regional studies suggest incremental improvements in some settings, for example, facilities in the FIND Integrated Biobank network have implemented solar backup systems to reduce outages (21), systematic updated data across all LMICs remain limited (17). Rural collection sites and facilities in low-income countries continue to face more acute challenges than their urban or upper-middle-income counterparts (16). Therefore, system designs have to prioritize resilience by implementing offline-capable systems that synchronize data upon reconnection (11). Appropriateness is also crucial, favoring technologies that correspond with local technical capacities and minimize reliance on scarce expertise, thereby lowering long-term maintenance costs (133, 134). The deployment of virtual biobanks and cloud-based platforms in sub-Saharan Africa demonstrates how these approaches improve data reach and address operational barriers in infectious disease surveillance (135, 136). Furthermore, the adoption of standardized digitization protocols serves to reduce the risk of creating heterogeneous data repositories, which often arise when disparate collection methodologies lack a unified quality control framework (28). AI represent a rapidly advancing frontier in digital infrastructure for biobanking in LMICs, warranting explicit consideration. AI-based tools can automate biospecimen quality assessment, detect pre-analytical deviations in real time, expedite genomic variant interpretation, and prioritize samples for sequencing based on research relevance (137). These functions currently require specialist personnel, which most LMIC biobanks are unable to sustain (138). Two aspects of AI adoption are particularly relevant for LMICs. First, in contexts with limited specialist analytical personnel, AI tools that operate on low-specification hardware or via cloud-based application programming interfaces (APIs) offer a potential cost-reduction strategy. Second, AI-based quality control tools can decrease pre-analytical error rates, directly addressing the 60–70% error burden reported in laboratory settings, which may be even higher in LMICs due to longer transport distances and more frequent cold-chain interruptions (37).

### 4.2 Dimension two: interoperability

Interoperability refers to the ability of distinct systems to exchange, interpret, and utilize information reliably within biobanking networks (51). Within the DIGS Model, interoperability acts as a foundational design principle across all biobanking activities. This strategy facilitates the uninterrupted integration of biospecimens with phenotypic, genomic, and clinical data. Achieving interoperability requires adopting standardized data formats, terminologies, and exchange protocols consistent with ISO 20387 (12). The MIABIS standard, developed within the BBMRI-ERIC pan-European biobanking network, provides a practical reference for minimum dataset requirements enabling cross-institutional biobank discoverability; while designed for an HICs context, its core metadata schema offers a technically accessible starting point for LMIC harmonization efforts (62). The use of unique identifiers enables dataset linkage, while comprehensive APIs support secure data sharing and collective work (139, 140). However, the direct adoption of standards from HICs in LMICs may not consider local health systems, disease priorities, and cultural practices, therefore diminishing relevance (141). Consequently, adaptation to local contexts is essential: initiatives in Latin America demonstrate the benefits of harmonized criteria for interoperable clinical data among regions, improving reproducibility and global research integration while maintaining local specificity (51). This dimension also acknowledges probable drawbacks to standardization, such as the diminished inclusion of indigenous knowledge (142). Therefore, combined models are recommended to balance global compatibility with regional distinctiveness (143).

### 4.3 Dimension three: governance

Governance encompasses the ethical, legal, and regulatory structures that guide the collection, utilization, and dissemination of biobanking data and biospecimens (105). In the DIGS Model, it works as a core element that interconnects with all other dimensions to promote responsible stewardship and fair results. In LMICs, resilient governance requires national data protection legislation that aligns with global standards and local realities, as exemplified by the BCNet frameworks (144). Compliance with the Nagoya Protocol represents a minimum international legal standard that LMIC biobanking governance frameworks have to incorporate: its requirements for prior informed consent, mutually agreed terms, and benefit-sharing certificates directly operationalize the fair governance principles the DIGS Model promotes (93). Ethical review boards are vital for scrutinizing intricate data-sharing arrangements (145). Community involvement mechanisms are crucial for fostering trust and facilitating informed choices (146). Operationally, the CARE Principles for Indigenous Data Governance provide the governance standard that unifies these requirements: they transform community involvement from a procedural obligation into a structural devotion to data sovereignty and fair benefit distribution (96). The model stresses equitable collaborations, urging LMIC researchers to spearhead data analysis and publication to avert exploitation, especially in partnerships with high-income counterparts. Governance structures must continue adaptable across time (147). Consequently, governance evolves from an impediment to a facilitator of ethical and secure data practices, curtailing risks like biopiracy and advancing benefit-sharing for at-risk groups (148). AI adoption in LMIC biobanking also introduces governance challenges that the D3 dimension must address explicitly. When biospecimen-derived data are used to train AI models, questions of intellectual property, benefit-sharing, and data sovereignty become acute (149). AI models trained predominantly on HIC-derived genomic datasets may perform poorly in LMIC populations, leading to diagnostic or prognostic recommendations that are systematically less accurate (150). The DIGS Model’s Governance dimension recommends that LMIC biobanks adopt AI-specific data use agreements that specify: that model outputs derived from LMIC genomic data may not be commercialized without explicit benefit-sharing provisions consistent with the Nagoya Protocol (93); that AI tools deployed in LMIC institutions are subject to algorithmic transparency requirements enabling local audit; and that AI governance frameworks are co-developed with participant communities in accordance with the CARE Principles (96). Without these governance structures, AI adoption risks reproducing the extractive dynamics that the DIGS Model is specifically designed to prevent.

### 4.4 Dimension four: skills

Skills are the abilities needed to design, run, and manage digital biobanking systems. The DIGS Model treats skill development as essential, since technology is not effective without skilled local staff. In LMICs, building these abilities means offering training that covers lab science, data management, bioinformatics, and ethics, such as the ISBER Biobanking 101/201 workshops. It is also important to create career paths that help keep talent in local institutions. However, as the following analysis shows, training alone is not enough. Without investment in ways to retain staff, skilled people often leave for other organizations instead of staying in LMIC health systems. Peer networks, mentoring, and leadership programs can help share knowledge and give LMIC researchers opportunities to lead projects, not just participate.

New programs, such as the ASLM and Africa CDC’s joint training on ISO 20387 standards planned for 2025–2026, aim to close gaps in infectious disease biobanking skills across African Union countries. If these programs go ahead as planned, they could make a big difference in the workforce. Still, their success will rely on steady funding, strong support from institutions, and clear career paths that help keep trained staff in local organizations (83, 138).

Still, the main issue is capacity-building extractivism, where training helps LMIC scientists gain skills, but those skills often end up in universities and organizations in wealthier countries instead of staying in LMIC research and health systems (83, 85, 88). For example, Ingenhoff and colleagues show that fair partnerships are hard to achieve when LMIC partners lack their own research infrastructure, and Koum Besson explains how funding and training in LMICs frequently reinforce knowledge systems that favor HICs (85, 88). In biobanking, this happens when trained experts move to genomics groups in wealthier countries, sometimes even those that funded their training, leaving LMIC institutions still without the expertise they need (82, 83).

The Skills part of the DIGS Model cannot just concentrate on technical training without additionally addressing deeper issues about who controls expertise. Addressing this requires structural changes at three levels. At the individual level, bonded scholarship and fellowship schemes, in which training funding depends on a set period of service within the sponsoring LMIC institution, can coordinate personal incentives with wider retention goals. Several African Union member states have tried these approaches in health workforce development, with promising early results (138). At the institutional level, LMIC-led training consortia, where curriculum design, quality assurance, and credentialing are managed by LMIC universities and professional bodies instead of being licensed from HICs, are key to building self-sustaining training infrastructure. The Africa Centres for Disease Control and the African Society for Laboratory Medicine are well placed to lead such consortia, as shown by their ISO 20387 training initiative (83, 138). At the international level, collaboration frameworks, which connect LMIC biobanking practitioners throughout regions without HICs involvement, offer another way to share knowledge and spread expertise more evenly, rather than concentrating it in high-income centers (43). Together, these organizational changes support the program-level efforts described above.

### 4.5 The interconnections in the DIGS Model

The efficacy of the DIGS Model stems from its attention to the interdependence among its dimensions. Digital infrastructure devoid of governance may foster extractive practices, undermining ethical soundness; governance absent skills yields unimplementable policies; skills without infrastructure result in underutilized human resources; and interoperability without these supports fragments data value. This linkage positions the model as a multifunctional diagnostic tool for assessing dimension-specific vulnerabilities in LMIC biobanks and a prescriptive blueprint for holistic interventions. By promoting simultaneous advances across several dimensions, the DIGS Model facilitates resilient biobanking frameworks that improve research equity, diagnostic development, and global health preparedness in LMICs. The DIGS Model is built on established frameworks related to global health, data science, and research governance. Charting these relationships recognizes prior scholarship and clarifies the particular contributions of the DIGS Model. Table 2 presents the relationship between DIGS and four key frameworks, summarizing its distinctive features.

**Table 2:** Comparison of the DIGS Model with four established frameworks in global health, data governance, and genomic research.

Framework	Primary focus	DIGS dimension addressed	Relationship to DIGS
WHO health system building blocks (152)	Comprehensive health system strengthening across six domains: service delivery, health workforce, information, medical products, financing, leadership/governance	Governance (leadership/governance); Skills (health workforce); digital infrastructure (information, medical products)	DIGS adapts and specifies these broad health system components for the specific context of biobanking, translating general principles into operational requirements for biospecimen-data ecosystems
FAIR data principles (151)	Technical guidance for making data findable, accessible, interoperable, and reusable	Interoperability; digital infrastructure	DIGS incorporates FAIR as a foundational technical standard but extends beyond data management to address governance structures and human capabilities necessary for FAIR implementation in resource-constrained settings
H3Africa framework (4, 94)	African-led genomic research governance with emphasis on capacity-building, community engagement, and data sovereignty	Governance; skills	DIGS builds on H3Africa’s pioneering model of equitable partnership, but generalizes beyond the African context while integrating explicit attention to digital infrastructure and interoperability that H3Africa addresses primarily through practice rather than as formal framework dimensions
GA4GH frameworks (95)	International standards for responsible genomic data sharing, including data use oversight and privacy-preserving technologies	Governance; interoperability	DIGS aligns with GA4GH technical and ethical standards but contextualizes them for LMIC settings where infrastructure constraints, regulatory gaps, and resource limitations require adaptation rather than direct application

First, the DIGS Model offers an integrated perspective, in contrast to present frameworks which deal with specific dimensions in isolation. Although each framework delivers important guidance, none offers a unified approach that integrates the four dimensions essential to biobanking in LMICs. The DIGS Model points out the need to develop digital infrastructure, interoperability, governance, and skills concurrently. This integrated approach is absent from frameworks that treat these areas separately and address them at different stages.

Second, the DIGS Model is specifically designed for resource-limited settings. Many present frameworks presuppose the infrastructure and governance context of HICs. For example, the FAIR Principles require reliable connectivity and technical expertise. Although the WHO building blocks have global relevance, they require significant adaptation for biobanking applications. The DIGS Model confronts the realities of LMICs by underscoring resilience, appropriateness, and adaptability.

Third, the DIGS Model places the relationship between biospecimens and data at its core. It addresses the “biospecimen-data divide” affecting biobanking in LMICs, providing a clear problem focus that broader frameworks lack. While the WHO building blocks may note weak health information systems, DIGS directly tackles the separation between physical biospecimens and digital data, which limits biobanking’s scientific and ethical value. This precise approach grants more efficient interventions.

Fourth, the DIGS Model includes equity within its structural design. Instead of treating equity as an add-on or checklist, DIGS integrates it throughout all dimensions. Fair digital infrastructure avoids reliance on resources unavailable in LMICs; equitable interoperability supports local priorities; fair governance ensures LMIC leadership over data control, along with equitable skills development, fosters local career pathways. This structural approach distinguishes DIGS from frameworks that address fairness mainly through procedural mechanisms such as consent or public engagement.

The DIGS Model brings together existing ideas and develops them to create new concepts. It combines technical perspectives like FAIR (151) and GA4GH (95), systemic approaches from WHO (152), and partnership views from H3Africa, which have frequently worked separately. The DIGS Model moves these frameworks forward by clearly showing how their different parts connect, adapting global standards for LMIC biobanking, focusing on the key link between biospecimens and data, and making equity a built-in part of the model instead of only a goal. By bringing together and building on existing ideas, the DIGS Model offers a framework created for the particular needs of biobanking and genomic research in LMICs. It gives researchers, funders, and decision-makers a common language and a tool to spot gaps and plan solutions, while keeping flexible as the field changes.

### 4.6 Operationalizing the DIGS Model: illustrative applications

The DIGS Model is a conceptual framework, not a step-by-step guide, but it can still be used as a practical diagnostic tool. Here are three examples that show how its four dimensions apply to real-world projects.

Rwanda’s eHealth system shows how D1 and D2 can work together. The Rwanda Health Management Information System links community health workers, district hospitals, and national labs on one digital platform. This lets biospecimen data from rural sites reach central repositories without needing to be entered again by hand (98). The example shows that investing in digital infrastructure (D1) only works well if you also set up interoperability standards and system architecture (D2) from the start. It also highlights a key DIGS point: upper-middle-income countries with steady political support for IT can reach interoperability levels similar to many HICs. In contrast, low-income countries with scattered, donor-driven investments still struggle with basic infrastructure.

The FIND Integrated Biobank Network illustrates the D3 dimension in practice. FIND’s partnership model requires equitable governance contracts: specifying data ownership, benefit-sharing arrangements, and LMIC co-authorship rights, as a condition of network participation (21). This transforms governance from a compliance exercise into a structural feature of the biobanking relationship. For institutions applying the DIGS Model diagnostically, FIND’s governance framework provides a template for assessing whether existing partnership agreements meet minimum equity standards.

The Africa CDC/ASLM ISO 20387 training initiative, targets the D4 dimension through competency-based workforce development in infectious disease biobanking across African Union member states (70, 126). Crucially, it pairs technical training with institutional anchoring mechanisms—requiring participating institutions to designate a trained biobank coordinator—which directly addresses the capacity-building extractivism problem described in Section 4.4. This illustrates how the Skills dimension of the DIGS Model must be implemented through institutional retention strategies, not just individual training events.

Together, these examples suggest diagnostic application: institutions and funders can map their existing activities against the four DIGS dimensions to identify which are being addressed and which are absent. A programme that invests heavily, e.g., in cold-chain equipment without corresponding data standards, governance agreements, or trained staff investment is, by the DIGS Model’s logic, likely to generate biospecimen collections that remain scientifically inert. This diagnostic use requires no quantitative scoring; it is primarily a structured checklist for strategic planning discussions.

## 5 Discussion

### 5.1 Implications for research and practice

The DIGS Model holds major implications for how biobanking and genomic research are carried out in LMICs. First, it confronts the common emphasis on physical infrastructure. Funders and governments have often chosen to invest in buildings, freezers, and lab equipment rather than digital infrastructure. The DIGS Model suggests this approach is incomplete. A biobank with strong physical resources but weak digital systems cannot support precision medicine; it becomes just an expensive freezer. To fully benefit from biospecimens, investments should focus more on digital aspects of biobanking. This includes making IT systems and workforce training a core part of project planning from the beginning, not something added later (117). Second, the DIGS Model encourages new forms of collaboration between institutions in HICs and those in LMICs. Previously, high-income institutions were viewed as the experts and technology providers, while LMIC institutions mainly provided biospecimens and clinical data. The DIGS Model recommends that real, long-term partnerships should help build digital skills in LMICs, so local researchers can lead in data assessment and governance. Third, the DIGS Model shifts our insight of sustainability. Previously, sustainability meant keeping freezers working and replacing equipment. Now, it involves developing the skills and systems needed to manage data over time. This includes training future bio-data stewards, building local governance structures, and creating systems that can adjust to new technologies and changing needs.

### 5.2 Reflections and limitations

Like other conceptual frameworks, the DIGS Model has some limitations that should be acknowledged. First, the DIGS Model has not yet been tested or validated with real-world data. It was developed by reviewing existing literature, not by collecting new data or conducting field tests. The model does not yet have tested indicators, a standard assessment tool, or published case studies showing its use in LMIC biobanking.

Second, the evidence base supporting the DIGS Model demonstrates both geographical and economic bias. Most case studies focused on low- and middle-income countries (LMICs) originate from sub-Saharan Africa. References show that African organizations and case studies from countries like Rwanda, Ethiopia, Nigeria, and Uganda make up most examples, while regions such as South and Southeast Asia, Latin America, the Pacific Islands, and the Middle East are less represented. Economically, the evidence base is concentrated in lower-income settings within the LMIC category. LICs and LMICs provide the majority of empirical cases, whereas UMICs are less systematically examined (153). Future empirical research validating the DIGS Model should prioritize upper-middle-income country case studies, particularly from Latin America and Southeast Asia, where biobanking practices are more advanced but governance and interoperability failures persist (51). Third, the DIGS Model’s digital focus may not suit some LMIC communities where non-digital, trust-based systems of biospecimen governance are more appropriate or culturally embedded. DIGS Model is intended for situations where digital integration is a goal.

The DIGS Model intends to contribute to the continuing discussion of making biobanking both beneficial and fair in resource-limited settings. This analysis underlines several areas for future research. Empirical studies should test how well the DIGS Model helps identify gaps and develop solutions. Comparing different LMIC settings could reveal how the model should be adapted for local conditions. Action research can explore how the model supports partnerships and builds capacity. Further ethical analysis could also strengthen the model’s focus on equity, justice, and sovereignty.

### 5.3 Conclusion

The gap between biospecimens and data is a major, yet commonly overlooked, barrier to realizing the benefits of genomic research for global health equity. By treating biobanking as a digital enterprise, the DIGS Model offers a way to understand and address this gap. It stresses that digital infrastructure, interoperability, governance, and skills should be developed together, not separately or in sequence. The model also challenges current investment priorities and partnership models in LMIC biobanking. It imagines a future where LMICs are not just sources of biospecimens but engaged participants and leaders in genomics.
